# Approaches to manipulating microRNAs in neurogenesis

**DOI:** 10.3389/fnins.2012.00196

**Published:** 2013-01-17

**Authors:** Haijun Zhang, Benjamin Shykind, Tao Sun

**Affiliations:** ^1^Department of Cell and Developmental Biology, Weill Medical College of Cornell UniversityNew York, NY, USA; ^2^Weill Cornell Medical College in QatarDoha, Qatar

**Keywords:** neurogenesis, microRNAs, miRNA inhibitor, miRNA sponge, mRNA protector

## Abstract

Neurogenesis in the nervous system is regulated by both protein coding genes and non-coding RNA molecules. microRNAs (miRNAs) are endogenous small non-coding RNAs and usually negatively regulate gene expression by binding to the 3′ untranslated region (3′UTR) of target messenger RNAs (mRNAs). miRNAs have been shown to play an essential role in neurogenesis, regulating neuronal proliferation, differentiation, maturation, and migration. An important strategy used to reveal miRNA function is the manipulation of their expression levels and patterns in specific regions and cell types in the nervous system. In this review we will systemically highlight established and new approaches used to achieve gain-of-function and loss-of-function of miRNAs *in vitro* and *in vivo*, and will also summarize miRNA delivery techniques. As the development of these leading edge techniques come online, more exciting discoveries of the roles miRNAs play in neural development and function will be uncovered.

The proper development of the nervous system relies on precisely programmed regulation of gene expression. The discovery of the non-coding **microRNAs (miRNAs)** has revealed a new paradigm of gene expression control, and the manipulation of miRNA expression levels and patterns in the nervous system has demonstrated the critical role they play in **Neurogenesis**. In this review, we will highlight various leading edge approaches that have been used to investigate miRNA function in neural development.

## miRNA discovery and biogenesis

miRNAs are endogenous, small non-coding, single stranded RNA molecules of ~22 nucleotide (nt) in length. Most miRNAs act as repressors of target messenger RNAs (mRNAs) by a posttranslational regulatory mechanism. Since the first identification of miRNA *lin-4* in *Caenorhabditis. elegans*, the database of published miRNA sequences has rapidly expanded, with 1600 miRNA precursors and 2042 mature miRNAs in humans, 855 precursors and 1281 mature miRNAs in mice, 238 precursors and 426 mature miRNAs in *Drosophila*, and 223 precursors and 368 mature miRNAs in *C. elegans* (The miRBase Sequence Database Release 19, http://www.mirbase.org) (Kozomara and Griffiths-Jones, 2011).

In most cases, miRNAs are transcribed as single-stranded primary miRNA (pri-miRNA) from intragenic or intergenic genomic regions by RNA polymerase II (Pol II) (Lee et al., 2004; Rodriguez et al., 2004). The pri-miRNAs are further cleaved by the RNase III-type nuclease Drosha and its co-factor DiGeorge Syndrome Critical Region Gene 8 (DGCR8/Pasha) to produce ~70 bp precursor miRNAs (pre-miRNAs) (Lee et al., 2003; Denli et al., 2004; Gregory et al., 2004). A different biogenesis pathway exists for miRNAs that are transcribed from intronic regions of protein-coding genes, termed mirtrons that are processed by the spliceosome and then by lariat debranchase activity to generate pre-miRNAs in various species (Berezikov et al., 2007; Okamura et al., 2007; Ruby et al., 2007). The hairpin shaped pre-miRNAs from both the canonical and mirtron biogenesis pathways are transported from nucleus to cytoplasm, and further cleaved by another RNase III member Dicer into imperfect complementary double stranded mature miRNAs of ~18–25 bp (Hammond et al., 2000; Grishok et al., 2001; Hutvagner et al., 2001). In addition, a number of alternative miRNA biogenesis pathways such as Drosha/DGCR8 independent and Dicer dependent miRNA biogenesis pathways have been reported (Yang and Lai, 2011). And the maturation of miR-451 has been shown to require argonaute 2 (Ago2) but not Dicer (Cheloufi et al., 2010; Cifuentes et al., 2010; Yang et al., 2010).

**Figure 1:**
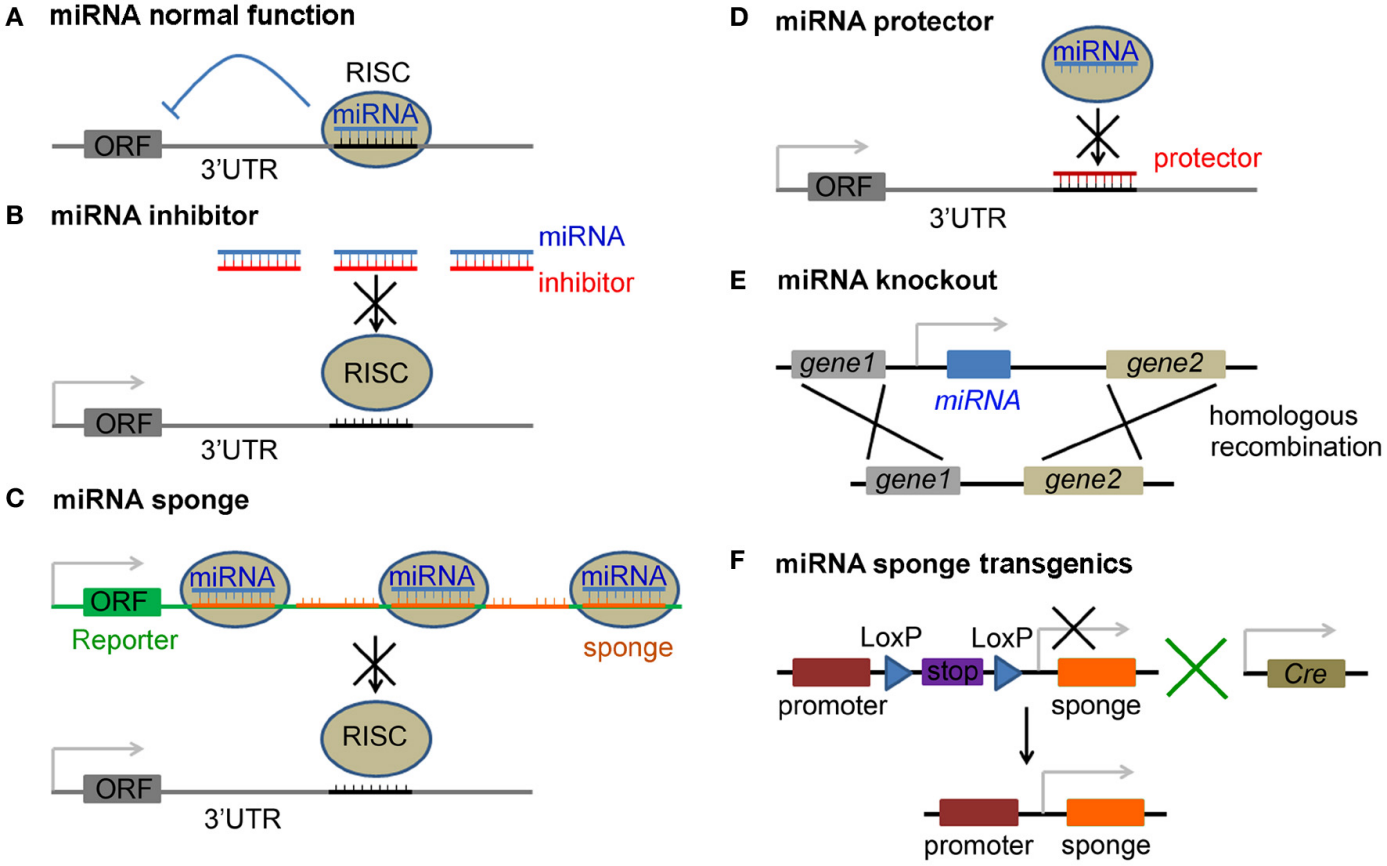
**Approaches to inhibition of miRNA function. (A)** Normal function of miRNAs is to suppress translation of the target mRNA with an open reading frame (ORF), or cause mRNA degradation, by guiding RNA-induced silencing complex (RISC) to the 3′ untranslated region (3′UTR) of the mRNA. **(B)** miRNA inhibitors are antisense miRNA oligonucleotides (AMOs), including 2′-O-methyl modified AMO, antagomir, locked nucleic acid (LNA), phosphorodiamidate morpholino oligonucleotide (PMO) and peptide nucleic acid (PNA), and block miRNA silencing activity by a complimentary binding to the mature miRNA. **(C)** miRNA is saturated by miRNA sponges that carry tandem multiplex of complementary sequences, which usually imperfectly match the target miRNA and are inserted in the 3′UTR of a reporter gene. **(D)** mRNA protector functions by a perfect binding to the 3′UTR of a mRNA and protects it from being bound by its miRNA. **(E)** Loss-of-function of a miRNA is achieved by direct miRNA knockout from the genome. **(F)** Tissue specific blockage of miRNA activity is achieved by breeding floxed miRNA sponge transgenic mice (with a stop signal flanked by two LoxP sites) with a proper Cre line.

For most miRNAs, one strand from the mature miRNA duplex is loaded into the RNA−induced silencing complex (RISC) (Hutvagner and Zamore, 2002; Chendrimada et al., 2005; Bartel, 2009). The RISC, guided by miRNA, binds to the 3′*UTR* of target mRNAs through specific complementarity of the 2–7 nt at the 5′$ end of the miRNA, which is termed the seed sequence (Lewis et al., 2003, 2005). Translation of mRNAs targeted in this manner is suppressed and the mRNAs may undergo degradation (Bartel, 2009) (Figure 1A). The RISC-miRNA pathway thus represents a novel and important gene silencing mechanism that exists in many organisms.

## Approaches to manipulating miRNAs in the nervous system

In the nervous system, the process of neurogenesis involves precise regulation of neuronal proliferation, differentiation, maturation, and migration. Accumulating evidence has highlighted the critical role that miRNAs play in neurogenesis (Kosik and Krichevsky, 2005; Kosik, 2006; Fineberg et al., 2009; Liu and Zhao, 2009; Lau and Hudson, 2010; Shi et al., 2010; Bian and Sun, 2011; Cochella and Hobert, 2012; Luikart et al., 2012). We here summarize approaches that have been used to manipulate miRNA expression in order to investigate their functions.

### Approaches to blocking miRNA biogenesis

miRNA biogenesis has been blocked in different regions or cell types in the nervous system by tissue specific ablation of *Dicer* using various Cre lines such as the *Emx1-Cre* line in the embryonic cortex and the *CamKII-Cre* line in the postnatal brain and adult hippocampus (Cuellar et al., 2008; Davis et al., 2008; De Pietri Tonelli et al., 2008; Kawase-Koga et al., 2009; Shin et al., 2009; Soukup et al., 2009; Andersson et al., 2010; Budde et al., 2010; Huang et al., 2010; Kawase-Koga et al., 2010; Konopka et al., 2010; Zehir et al., 2010; Zhao et al., 2010b,c; Zheng et al., 2010; Iida et al., 2011; Li et al., 2011; Liu et al., 2011; Nowakowski et al., 2011; Tao et al., 2011; Chen and Wichterle, 2012; Repetto et al., 2012; Rosengauer et al., 2012). Mice with tissue specific Dicer deletion show various neural defects, suggesting an important role of miRNAs in neural development and function. Moreover, a mouse model with a microdeletion syntenic to the human chromosome 22q11 deletion, which includes DGCR8 gene, has shown altered miRNA biogenesis, suggesting a role of miRNAs in behavioral and cognitive deficits in humans with 22q11 deletion (Stark et al., 2008). Postmitotic neurons in the brain have shown more severe defects in Dicer knockout mice than DGCR8 knockout mice, which are both ablated by the *CamKII-Cre* line, suggesting that a subpopulation of miRNAs generated by Dicer but bypassed by DGCR8 processing may play an important role in postmitotic neuron development (Babiarz et al., 2011). In *Drosophila*, Dicer1 and Pasha mutations but not Ago1 and Ago2 mutations have shown disrupted olfactory projection neuron phenotypes (Berdnik et al., 2008).

Ablation of molecules in the miRNA biogenesis pathway has proven important roles of miRNAs in neural development. However, the weakness of such studies should also be noticed. First, a large number of miRNAs is affected when miRNA biogenesis is blocked, making it critical to distinguish the role of individual miRNAs in neurogenesis. Second, Dicer is also required for maintaining the heterochromatin assembly, likely by the short interfering RNA (siRNA) pathway, and cleaving long strand Alu transcripts (Fukagawa et al., 2004; Kanellopoulou et al., 2005; Yang and Lai, 2011). Thus, phenotypes of Dicer deletion need to be carefully interpreted.

### Approaches to overexpression of miRNAs

#### miRNA mimics

Gain-of-function for a specific miRNA can be achieved by overexpressing the mature sequence of the miRNA. miRNA mimics can be chemically synthesized as oligonucleotides according to sequences of the endogenous miRNA. Double stranded miRNA mimics, with the sequence of one strand identical to the endogenous mature miRNA, are usually used to increase the efficiency of augmenting miRNA expression. The strand identical to the endogenous miRNA will be loaded into the RISC complex and silence target genes as the endogenous (Martinez et al., 2002). When designing a miRNA mimic, one needs to be cautious to avoid formation of a new miRNA from the complementary strand of the mimic duplex. Since there is no additional sequence introduced in the target system, the synthesized miRNA mimic has no vector-based toxicity. Studies have shown that infusion of miR-132 mimics to the mouse visual cortex blocks ocular dominance plasticity produced by monocular deprivation (Tognini et al., 2011), and miR-137 mimics negatively regulates neural stem cell proliferation (Sun et al., 2011). However, the transfection efficiency of miRNA mimics is low, especially in neurons, and the transfection is usually transient, which has limited its application.

#### miRNA precursors

A vector-based technique has been used to obtain a long-term stable expression of miRNAs by inserting the miRNA precursor sequence downstream of the RNA polymerase III (Pol III)-driven or Pol II-driven promoters in a vector system (Takamizawa et al., 2004; Chung et al., 2006; McLaughlin et al., 2007). Plasmid and virus-based overexpression of miRNA precursors allows ectopic expression of a miRNA in specific regions and cell types in the nervous system, for instance introduction of the miR-9 precursor plasmid in spinal motor neurons and the miR-124 precursor in mouse subventricular zone stem cells (Cheng et al., 2009; Otaegi et al., 2011b). However, the amount of overexpression needs to be carefully optimized to minimize side effects, for example consistent expression of miRNAs and large amount of short hairpin RNAs (shRNAs) can induce serious liver injury and lead to mouse lethality due to oversaturation of the endogenous small RNA pathways (Grimm et al., 2006).

#### miRNA biogenesis enhancement

Instead of manipulating miRNAs themselves, miRNA expression levels can be modified by modulating the miRNA processing pathway. The fluoroquinolone antibiotic, enoxacin, has been found to promote the biogenesis of endogenous miRNAs. Among the upregulated miRNAs, a number of them such as miR-124a, miR-125a, miR-23a, and let-7b are highly involved in neural development (Shan et al., 2008). Moreover, enoxacin has been shown to enhance the production of tumor suppressor miRNAs and miRNAs associated with neurogenesis such as let-7, miR-125, and miR-7 by binding to the miRNA biosynthesis protein TAR RNA-binding protein 2 (Melo et al., 2011). To manipulate biogenesis of individual miRNAs, small molecule libraries have been screened to identify a miR-122 activator in hepatocellular carcinoma cells (Young et al., 2010). While mechanisms by which small molecules activate miRNA expression are largely unclear, these studies have allowed development of novel tools to manipulate miRNA expression such as screening small molecules for activating miRNA expression in neurogenesis.

The gain-of-function approach by overexpressing miRNAs has both advantages and disadvantages (Table 1). miRNA mimics can be directly synthesized and delivered, making them convenient to use. Plasmid or virus based miRNA precursors are easy for delivery in tissues and cells that miRNA mimics are difficult to introduce such as neurons. However, overexpression of miRNAs may cause side effects such as oversaturation of the miRNA processing pathway. The efficiency of processing mature miRNAs from their precursors can vary and may affect their function. Using miRNA precursors flanking with longer genomic DNA sequences can improve the efficiency miRNA processing. The miRNA biogenesis enhancement strategy using small molecules is easy to administrate *in vitro* and *in vivo*, and is able to elevate levels of multiple miRNAs simultaneously, but it still requires optimization to be able to elevate specific miRNAs. Nevertheless, overexpression of miRNAs is a powerful approach to manipulate miRNAs in neural development.

**Table 1:** **Advantages and drawbacks of miRNA gain-of-function technologies**.

**Technology**	**Advantages**	**Drawbacks**
miRNA mimics	Convenient; time saving	Expensive; non-specific effect; difficult to introduce into neurons, toxicity
miRNA precursors	flexible for *in vitro* and *in vivo* studies	Unpredicted processing efficiency; vector-based side effect; toxicity for mass overexpression; non-specific effect
miRNA biogenesis enhancement	Small molecules, easy to administrate; upregulating multiple miRNAs simultaneously	Unknown tissue distribution; unclear mechanisms; low miRNA specificity

### Approaches to knockdown of miRNAs

#### miRNA inhibitors

Antisense miRNA oligonucleotides (AMOs) have been used as **miRNA inhibitor** to knock down endogenous miRNA activity. The sequences of these oligonucleotide analogs are complementary to the endogenous mature miRNAs, and function by directly binding to the single strand mature miRNA to block miRNA silencing (Figure 1B). We here highlight inhibitor technologies developed to block miRNA silencing activities.

***2′-O-methyl group modified AMOs***. Native oligonucleotides are sensitive to degradation in serum or by endogenous cellular exonucleases and endonucleases, and their efficiency to cross cell membranes is low, making them ineffective in *C. elegans* (Hutvagner et al., 2004). Therefore, chemically modified AMOs has been generated. 2′-O-methyl group modified AMOs have been shown to significantly increase resistance to nuclease degradation, display enhanced binding to miRNAs and inhibit endogenous miRNA activities (Hutvagner et al., 2004). For example knocking down let-7b and miR-124 using 2′-O-methyl AMO can promote proliferation of neural stem cells (Cheng et al., 2009; Zhao et al., 2010a). Although 2′-O-methyl group modified AMOs show longer survival time in the intracellular environment than common DNA oligonucleotides, they are still unstable in serum (Lennox and Behlke, 2010). Since nuclease degrades nucleic acids by breaking the phosphodiester bonds between nucleotides, replacing these bonds by phosphorothioate linkage in AMOs has been effective in reducing degradation. The drawback of this modification, however, is that it decreases the binding of the AMO to its target miRNA.

***Antagomirs***. Antagomirs are 22–23 nt 2′-O-methyl modified, 3′ end cholesterol-conjugate RNA analogs that have a complementary sequence to a miRNA. Some phosphodiester linkages in the phosphate backbone of **Antagomir** are substituted by phosphorothioate and the cholesterol conjugation facilitates the *in vivo* delivery of this AMO. Studies have shown that the antagomir for miR-122 exhibits specific inhibition of miR-122 in the mouse liver by tail vein injection, and the antagomir for miR-16 inhibits miR-16 expression in multiple tissues, including direct injection of miR-16 antagomir into mouse brains (Krützfeldt et al., 2005, 2007). The advantage of antigomirs is that they are nuclease resistant, and can be delivered into cells directly without any vector assistant, which avoids complication of using delivery vehicles. The drawbacks of antagomirs are high usage dose and possible off-target effects, which has limited their application as a therapeutic reagent in humans (Krützfeldt et al., 2005; Morrisey, 2010; Patrick et al., 2010).

Other 2′ site modifications of AMOs have been developed to produce more powerful miRNA inhibitors. Introduction of 2′-O-methoxyethyl groups increases affinity and specificity of binding to miRNAs compared to 2′-O-methyl analogs (Hutvagner et al., 2004). Moreover, AMOs with both 2′Flouro (2′F) and phosphorothioate backbone modifications have been shown to display high efficiencies of inhibition of miRNAs, with an effect further enhanced by addition of 2′-O-methoxyethyl groups to both ends (Davis et al., 2006, 2009).

***Locked nucleic acids (LNAs)***. In the LNA molecule, the 2′-O and 4′-C of the ribose are bridged by a methylene group, which results in a bicyclic nucleotide with a locked conformation. This specially locked structure stabilizes the LNA/RNA duplex and makes them strongly resistant to nuclease degradation (Kaur et al., 2006). LNA probes have been extensively used to detect spatiotemporal miRNA expression, in such techniques as Northern blot analysis (Valoczi et al., 2004), *in situ* hybridization (Wienholds et al., 2005; Kloosterman et al., 2006; Nelson et al., 2006) and miRNA expression profiling assays (Castoldi et al., 2006). In addition to their use as probes, LNA-based miRNA inhibitors have been designed by forming an LNA core sequence with flanking DNA sequences, generating a DNA-LNA-DNA chimeric molecule. The DNA-LNA-DNA oligonucleotides have been shown to inhibit activities of miR-21 in glioblastoma cell lines (Chan et al., 2005), and miR-181 in the differentiation process of mouse myoblasts (Naguibneva et al., 2006). Moreover, LNA-only AMOs can suppress miRNA *bantam* in cultured *Drosophila* cells (Orom et al., 2006). Phosphorothioate modified LNA-based miR-122 inhibitor can markedly reduce miR-122 silencing activities at lower dosages compared to the miR-122 antagomir (Krützfeldt et al., 2005; Elmén et al., 2008). Inhibiting miR-219 by intracerebralventricular deliver of LNA AMO can increase its target gene expression in prefrontal cortex (Kocerha et al., 2009). Because of their high efficiency blocking of miRNA activities, LNA-based AMOs have been developed for use as therapeutic tools: an LNA-based miR-122 AMO is under phase II clinical trail for anti-hepatitis C therapy (Lanford et al., 2010).

***Phosphorodiamidate morpholino oligonucleotides (PMOs)***. PMOs are designed as a strategy in which the riboses of the nucleic acid are substituted by 6-membered morpholine rings, and the phosphodiester bonds are substituted by phosphorodiamidates to produce stronger steric blocking of nucleases and prevent degradation. miR-124 has been shown to regulate muscle cell fate in zebrafish by knocking down miR-124 activity using PMOs (Flynt et al., 2007). Microinjections of PMOs targeting miR-183 family members in zebrafish embryos have demonstrated their roles in sensorineuron fate determination (Li et al., 2010). To improve bioavailabilities, further modifications of PMOs have been developed, called vivo-Morpholinos, which show more efficient penetration in multiple tissues *in vivo* (Morcos et al., 2008).

***Peptide nucleic acids (PNAs)***. PNAs are artificial DNA/RNA mimics that have peptides flanking the nucleic acid sequences to increase target affinity, specificity, nucleases resistance and/or cell penetration. Delivering PNAs directly by either electroporation or conjugation of PNA with cell penetrating peptides, or by linkage of PNAs with four lysine residues has demonstrated inhibition of miR-122 activity in human liver cancer cells (Huh7) and rat primary hepatocyte cells with high efficiency (Fabani and Gait, 2008). PNA AMOs has also been shown to inhibit miR-155 function in mice *in vivo* (Fabani et al., 2010). By screening 11 cell-penetrating peptides, Oh et al., have shown that a Tat-modified-conjugated PNA is the most effective for delivering into cells and inhibiting miRNA function without the assistant of transfection reagents (Oh et al., 2010). PNA AMOs delivered into neurons have been shown to suppress miR-326 activities and cause an increased expression of a synaptic plasticity-related gene Arc, indicating a promising approach in neuroscience research (Wibrand et al., 2012).

The effects of AMOs need to be carefully interpreted. Some studies have shown anti-heart failure effect of miR-21 antagomirs, but some have shown no effect, which is also supported by genetic deletion study of miR-21 (Thum et al., 2008, 2011; Morrisey, 2010; Patrick et al., 2010). The difference is likely caused by high concentration of delivered antagamirs that can bind to target tissues and induce non-specific disruptions of gene expression. The delivery methods for antagomirs such as vein injections may interfere with tissue distribution and metabolisms of AMOs, or cause toxicities. Optimizing AMO design will reduce the amount of AMO usage *in vivo*, and in turn reduce AMO toxicity and off-target effects (Park et al., 2011).

#### miRNA sponges

The transient nature of current AMO miRNA inhibitors has driven the development of new approaches, such as **miRNA sponge**, to achieve long-term miRNA loss-of-function. The miRNA sponge contains multiple binding sequences complementary to a mature miRNA and can bind to endogenous miRNAs and block their silencing activity (Figure 1C). The most common miRNA sponge design involves the insertion of a tandem multiplex artificial miRNA binding sequence into the 3′UTR of a Pol II-driven reporter gene, such as green fluorescence protein (GFP) or luciferase. The miRNA binding sequence of the sponge can be perfectly complementary to the seed sequence of the miRNA and then bulged, or kept complementary at positions 9–12 (Ebert et al., 2007; Gentner et al., 2009; Loya et al., 2009). The bulge is constructed on purpose to protect against RNA interference-type cleavage and degradation of the sponge RNA by the Ago2 component of the RISC. Sponges with exact complementary to the target miRNA also have been shown to have inhibitory effects (Ebert et al., 2007; Ebert and Sharp, 2010). In addition, there is a second miRNA sponge system that utilizes the Pol III promoter to drive sponge expression, in which 5′ and 3′ stem-loop structures are added to stabilize the RNA products to substitute the reporter gene in the Pol II promoter system. Both Pol II- and Pol III-driven sponges with bulged or perfect miR-20 binding sites have been shown to successfully rescue miR-20 binding to its target sites (Ebert et al., 2007). An advantage of using the sponge approach is that binding sequences for a family of miRNAs (due to the conserved seed sequences) or even completely unrelated miRNAs can be constructed together in one vector. The silencing activities of these miRNAs can be repressed simultaneously to facilitate efficient suppression of multiple miRNAs.

A miR-9 sponge has been shown to modify motor neuron subtypes in the developing spinal cord (Otaegi et al., 2011a). By optimizing the sponge design, Otaegi et al., have shown that a shorter spacing between binding sequences and the inclusion of a coding gene improve the sponge effect. Moreover, optimizing the number of binding sequences also affects sponge activity (Otaegi et al., 2011b).

Similar to the sponge, tough decoy (TuD) RNA driven by RNA Pol III promoter (U6) has been developed to achieve long term suppression of miRNAs using lentiviral based vectors (Haraguchi et al., 2009). TuD RNAs containing two miRNA binding sites with four extra nts inserted in each and flanked by 3 nts linkers and two stem structures, show the strongest miRNA inhibition (Haraguchi et al., 2009). The use of recombinant adeno-associated virus (rAAV) to deliver the TuD RNAs for let-7 and miR-122 has shown strong, long term inhibition of let-7 and miR-122 expression in mice (Xie et al., 2012).

The miRNA sponge and TuD RNA techniques have shown promising inhibition of miRNAs *in vitro* and *in vivo*. However, the design and efficiency of sponge and TuD RNAs, as well as their delivery by either plasmid or viral vectors, require careful examination for individual miRNAs in the nervous system.

#### mRNA protectors

The approaches described above are focused on direct manipulations of miRNAs, which may affect expression of all target genes of these miRNAs. To evaluate the direct silencing activities of miRNAs on their specific target mRNAs, the **mRNA protector** technique has been developed. The mRNA protector has a perfect complementary sequence to the miRNA binding site on the 3′UTR of a putative target mRNA and can compete with endogenous miRNAs for the binding site, with higher affinity to prevent miRNA/RNA association (Figure 1D). For example, Choi et al., have used a PMO-based target mRNA protector *in vivo* to prevent the action of miR-430 on its target genes *squint* and *lefty* in zebrafish (Choi et al., 2007). Another morpholino target protector has been used to identify *hairy1* as a primary target for miR-9 in neural progenitors in *Xenopus* (Bonev et al., 2011). Current mRNA protectors using morpholinos are designed to bind to the region of the target mRNA complementary to the miRNA seed sequence and to 3′ or 5′ flanking sequences in the 3′UTR (Choi et al., 2007; Bonev et al., 2011; Staton and Giraldez, 2011). Whether oligo-based mRNA protectors have a long term effect *in vivo*, and the challenge of delivering mRNA protector oligos especially in mammals have limited their application. Developing a plasmid- or virus-based mRNA protector strategy is an option to achieve long-term and tissue specific exogenous protector expression. Moreover, as a miRNA often functions to regulate multiple target genes, protecting only one target mRNA may not reveal the full miRNA function. Nevertheless, due to the specificity of mRNA protectors, they can be used to screen major targets for a miRNA in neurogenesis.

#### Small molecule inhibitors

Small molecules serving as inhibitors of miRNAs have been explored. Using a luciferase-based assay, azobenzene was identified to selectively inhibit miR-21 transcription from more than 1000 compound screened (Gumireddy et al., 2008). Studies have demonstrated that inhibitors for miR-122 show specific blocking activity on miR-122 but no other miRNAs, suggesting that small molecules can be used as inhibitors for specific miRNAs (Young et al., 2010; Connelly et al., 2012). These data further indicate the feasibility of using small molecules to inhibit miRNA function in neurogenesis.

## Delivery techniques to manipulate miRNAs *in vivo*

The success of manipulating miRNA expression in the nervous system relies on efficient delivery systems. We here highlight the delivery techniques that can be used for examining miRNA function in neurogenesis.

### Microinjection

Microinjection is one of the simplest methods for gene delivery. miRNA mimics or AMO inhibitors can be injected into the yolk or cytoplasm of early embryonic cells, using fine glass needles with the assistant of a pressure microinjector and micromanipulator, to interrupt miRNA function. For example, microinjection of miR-124a precursors at the 8-cell stage revealed the role of miR-124a in early eye development in *Xenopus*, and double-stranded miRNA *let-7* was injected into the zygotes of zebrafish and frogs (Kloosterman et al., 2004; Qiu et al., 2009). Injections of miR-183, miR-96 and miR-182 LNA inhibitors in zebrafish embryos have demonstrated the roles of these miRNAs in the development of sensorineurons (Li et al., 2010). The miRNA microinjection technique was also applied to study the development of *Drosophila* (Berry et al., 2009). However, microinjection in mammalian embryos and postnatal tissues is difficult, especially in the nervous system, which has limited its application.

### Virus infection

Virus infection is a common tool to transport exogenous transgenes into target cells and tissues *in vitro* and *in vivo*. Adenoviral (Ad) vectors infect both dividing and non-dividing cells and can be used *in vitro* and *in vivo* (Xia et al., 2002; Xu et al., 2011). However, Ad vectors cannot integrate into the host genome, and are found to elicit the immune response, which has limited their use for long term studies of miRNA effects (Bessis et al., 2004). Adeno-associated viruses (AAVs) work in both dividing and non-dividing cells and unlike the Ad vector, the AAV does not induce strong immune reaction and can integrate into the host genome (Rutledge and Russell, 1997; Grimm and Kay, 2003). The AAV carried miRNA-based hairpins have been shown to efficiently knock down the photoreceptor-specific gene Peripherin-2 in the mouse retina (Georgiadis et al., 2010). miR-183/96/182 sponges in the AAV have been shown to inhibit activity of this cluster in the photoreceptor of mouse eyes *in vivo* (Krol et al., 2010). Retroviral vectors (RVs) including the lentiviral vectors (LVs) are genome-integrating vectors. The lentiviruses can express a gene of interest in both dividing and non-dividing cells, stem cells, zygotes and their differentiated progeny, to maintain persistent gene expression (Rubinson et al., 2003). Mice infected by miR-326 or its sponge LVs have been used to examine the pathogenesis of multiple sclerosis (Du et al., 2009). Studies have used RVs and LVs to overexpress and knock down miR-124 in mice, and demonstrated miR-124 function in postnatal and adult neurogenesis (Cheng et al., 2009; Åkerblom et al., 2012).

Virus infection has the potential to target cells and tissues other than the nervous system and causes side effects when delivered. Some viruses such as RVs and LVs may exhibit unexpected exogenous gene transcriptional silencing, which hinders highly efficient transgene expression. These are the factors that should be considered when using virus infection to study miRNA function in neurogenesis.

### *In utero/in ovo* electroporation

To conduct quick **Gain-of-function and loss-of-function** studies for a gene of interest in the developing nervous system, approaches such as *in utero* or *in ovo* electroporation have been widely used. This technique utilizes electric field pulses to drive nucleic acid molecules through pores generated in the cell membrane to introduce the exogenous transgene into cells. The *in utero* electroporation technique allows passage of the target gene plasmid DNA into the lateral ventricle of mouse embryonic brains through the uterus wall of the pregnant mouse, and introduction of DNA constructs into proliferative ventricular zone progenitor cells (Nishimura et al., 2012; Pacary et al., 2012). For example, functions of a brain-enriched miR-137 in the developing mouse brain have been tested using *in utero* electroporation (Sun et al., 2011). *In utero* electroporation of miR-9 and let-7 into embryonic mouse brains was used to study neural stem cell fate determination (Zhao et al., 2009a, 2010a). The role of miR-9 in motor neuron subtype specification has been examined in the chick embryonic spinal cord using *in ovo* electroporation (Otaegi et al., 2011a,b). Inhibition and overexpression of miR-124 by *in ovo* electroporation were applied to explore its function in chick neural tube development (Cao et al., 2007; Visvanathan et al., 2007). Studies have reported successful expression of an exogenous gene in primitive streak stage embryos using electroporation (Yasuda et al., 2000; Kobayashi et al., 2002). Electroporation may cause variations of expression levels and positions of an exogenous gene in neural tissues, and a large number of samples are required to draw a reliable conclusion. Nevertheless, *in utero*/*in ovo* electroporation is a powerful tool to manipulate miRNA expression in specific regions in the nervous system.

### miRNA transgenic animals

Although the procedure to construct transgenic animals is time consuming, it is still an effective strategy to test miRNA functions *in vivo*. miRNA gain-of-function can be achieved by miRNA overexpression transgenic constructs and loss-of-function can be achieved in miRNA gene knockout or sponge expression animals. Similar to coding genes, miRNAs can be ablated from the genome using DNA recombination, and also depleted in specific tissues via, for instance the Cre-LoxP system, to generate miRNA conditional knockout mice or tissue specific miRNA sponge transgenic mice (Figures 1E,F).

To study the function of miR-132 in hippocampal-dependant learning and memory, a doxycycline-regulated miR-132 mouse line has been generated to show activity-dependant regulation of miR-132 in cognition (Hansen et al., 2012). Conditional over-expression of miR-9^*^ and miR-124 under the control of the *nestin* promoter in neural progenitors has been shown to suppress their target gene BAF53a and lead to progenitor proliferation defects (Yoo et al., 2009). Transgenic mice with a lymphocyte specific miR-17–92 overexpression construct developed lymphoproliferative disease and autoimmunity and died prematurely (Xiao et al., 2008).

miRNA knockout mice have been used to reveal many aspects of miRNA functions (Park et al., 2010). Functions of miR-208 in heart growth, miR-155 in immune system and miR-1-2 in heart development have been demonstrated in miRNA knockout mice (Rodriguez et al., 2007; Thai et al., 2007; Van Rooij et al., 2007; Zhao et al., 2007). Knockout mice of the miR-17-92 cluster have revealed their critical function in embryogenesis (Ventura et al., 2008). miR-133 knockout mice have proven subtle function of miR-133 in midbrain dopaminergic neuron development (Heyer et al., 2012). In the double knockout mice in which *miR-9-2* and *miR-9-3* precursors are ablated, the brain development is severely affected due to proliferation defects in neural progenitors (Shibata et al., 2011). Conditional deletion of the miR212/132 locus using retrovirus-based Cre expression has shown dendrite growth and arborization defects in adult hippocampal neurons (Magill et al., 2010). In other species, the gene knockout technique is also utilized to explore the function of miRNAs in *C. elegans* and *Drosophila* (Clark et al., 2010; Sun et al., 2012).

Mature miRNA normally has several precursors with similar seed sequences and other miRNA precursors may functionally compensate the knockout of a single miRNA precursor, unless all precursors are ablated. For example, less severe phenotypes of miR-182 ablation in retina are likely caused by functional redundancy (Jin et al., 2009). miRNAs are often localized closely as clusters in the genome. Ablation of a single miRNA without disturbing other miRNAs is technically challenging. Generating sponge transgenic animals can overcome these drawbacks (Figure 1F). miRNA sponge transgenic mouse model that blocks the activity of the miR-183/96/182 cluster simultaneously and selectively in the retina has revealed their important role in acute light-induced retinal degeneration (Zhu et al., 2011). miR-29 sponge transgenic mice have demonstrated the role of miR-29 in suppressing intracellular pathogen-induced immune responses (Ma et al., 2011). Moreover, fly miRNA sponge transgenics has revealed the function of miR-8 in neuromuscular junction formation (Loya et al., 2009).

### Liposome, polymer, hydrogel, microsphere, and nanoparticle

A recent study has shown that liposome encapsulated plasmids or oligonucleotides can pass into the phospholipid bilayer, and cationic liposome protects oligonucleotides from nuclease degradation and facilitates cell uptake (Zhao et al., 2009b). However, liposome reagents are usually toxic, initiate immune responses, prone to accumulate in the reticuloendothelium system and have short half-life, all of which have limited their applications (Chiarantini et al., 2005; Aagaard and Rossi, 2007; Zhao et al., 2009b). Alternatively, a study using lipid-like materials called lipidoids to transfer 2′-O-methyl AMOs into cells has been proven to be safe and efficient in mice, rat and non-human primates models (Akinc et al., 2008). Moreover, biodegradable polymers are a solution to the problems of using liposomes. Polymer delivery of oligonucleotides has been shown to exhibit sustained release and better tissue distribution (Chirila et al., 2002). Hydrogels, microspheres and nanoparticles have also been evaluated for their suitability to transport nucleic acids into target cells and have been proved to be efficient, stable and of low toxicity (Rosi et al., 2006; Bisht et al., 2008; Zhao et al., 2009b). Moreover, nanoparticles have potential to allow the manipulation of miRNA expression by using cell type specific targeting molecules, such as peptides, ligands, antibodies and other bioactive molecules in neuronal cells. For example, increased accumulation of miR-124 has been detected in brains of mice with tail vein injection of a miR-124 nanocarrier (Hwang Do et al., 2011).

## Conclusions

Accumulating evidence has demonstrated the importance of miRNAs in neural development and function. Both gain-of-function and loss-of-function approaches have been applied to uncover the role of miRNAs in neurogenesis. We here have systematically summarized new techniques used to manipulate miRNA expression *in vitro* and *in vivo* and each approach has technical advantages and disadvantages. Combination of multiple approaches may be necessary to further advance the investigation of miRNA functions. Dysregulation of miRNAs has been found to be associated with several neurological disorders (Bian and Sun, 2011; Fiore et al., 2011; Sibley and Wood, 2011; Junn and Mouradian, 2012; Saito and Saito, 2012; Tan et al., 2012). Taking advantage of miRNA synthesis and delivery techniques, miRNA manipulations are becoming a promising means of gene therapy to treat human neurological diseases.

### Conflict of interest statement

The authors declare that the research was conducted in the absence of any commercial or financial relationships that could be construed as a potential conflict of interest.

## Key Concept

microRNAs (miRNAs): ~22 nucleotide endogenous small non-coding RNAs that exist in many organisms such as plants, invertebrates and vertebrates. Mature miRNAs regulate gene expression by binding to the 3′untranslated region (3′*UTR*) of target protein coding genes, and affect stability of messenger RNAs (mRNAs) and/or reduce protein translation.
Neurogenesis: A process of generating neurons from neural stem cells and neural progenitors. It involves tightly controlled regulation of proliferation, differentiation, migration and maturation.
miRNA inhibitor: Chemically synthesized antisense miRNA oligonucleotides designed to knock down endogenous miRNA silencing activity by directly binding to the single strand mature miRNA using complementary sequences.
Antagomir: A form of miRNA inhibitors that is a 22–23 nucleotide, 2′-O-methyl modified, 3′ end cholesterol-conjugate RNA analog with a complementary sequence to a mature miRNA.
miRNA sponge: An RNA transcript contains binding sites designed to be targeted by a specific miRNA or a family of miRNAs to block the silencing activity of the endogenous miRNAs to their putative targets.
mRNA protector: An RNA transcript or oligonucleotide contains a perfect complementary sequence to the miRNA binding site on the 3′UTR of a putative target mRNA. It competes with endogenous miRNAs for the binding site and prevents miRNA/RNA association to block the endogenous miRNA silencing activity.
Gain-of-function and loss-of-function: Approaches to altering function of a coding gene or a non-coding RNA by increasing and decreasing its expression levels in specific cells or tissues, respectively.
